# Management of cracked tooth using simvastatin as intracanal medicament

**DOI:** 10.1002/ccr3.3315

**Published:** 2020-09-17

**Authors:** Mohamed Fawzy, Hatem A. Alhadainy, Muhammad Salah‐Uddin, Saleem Abdulrab

**Affiliations:** ^1^ Department of Endodonics College of Dentistry Tanta University Tanta Egypt; ^2^ Madinat Khalifa Health Center Primary Health Care Corporation Doha Qatar

**Keywords:** cracked tooth, intracanal medicament, simvastatin

## Abstract

Cracked tooth syndrome is presented as pain associated with biting and sensitivity. Intracanal medication with simvastatin stimulates hard tissue formation at crack line, and the tooth was functioning on the follow‐ups.

## Graphical abstract

Cracked tooth syndrome is presented as pain associated with biting and sensitivity. Intracanal medication with simvastatin simulates hard tissue formation at crack line, and the tooth was functioning on the follow‐ups.

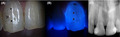

## 1 INTRODUCTION

Cracked tooth syndrome is a common well‐documented condition that may occur due to morphologic, physical, and iatrogenic factors. Epidemiologic data revealed that splits or fractures are the third most common cause of tooth loss, indicating a high clinical significance of this syndrome.^1^

Diagnosis of cracked tooth is challenging, and the treatment has been controversial. Stainless steel bands were often used as a diagnostic tool and a temporary before a full coverage restoration.^1^ Root canal treatment (RCT), followed by a crown, is recommended if the pulp becomes irreversibly inflamed.^2^ One study reported successful treatment with a bonded composite restoration after 6 months, with no differences between restorations with or without cusp coverage.^3^ The prognosis of the relevant tooth depends on the extent of the crack and whether the crack has extended through enamel, dentin, pulp, and/or the pulp chamber floor. Cracks that extended to the pulpal floor or beyond alveolar bone have been deemed hopeless.^2^

Simvastatin is a drug used primarily to treat hyperlipidaemia and protect against cardiovascular diseases. It has been shown to possess pleiotropic effects such as antimicrobial, anti‐inflammatory, immunomodulatory, antioxidant, and bone‐forming properties. Statins also exert an effect on dentin and pulp regeneration.^4^ A recent systematic reviews and meta‐analysis studies^5^, ^6^ indicated that adjunctive use of locally delivered statins to mechanical periodontal treatment is beneficial to increasing bone fill percentage and improved intrabony defects.

Data involving management of a cracked teeth are rare in the literature, which makes clinical decisions more difficult, and additional information regarding cracked teeth would provide a better perspective on the clinical management and outcome of these teeth. In this case report, we introduce a description for the treatment of a cracked maxillary central incisor using simvastatin as intracanal medicament.

## 2 MATERIALS AND METHODS

**FIGURE 1:**
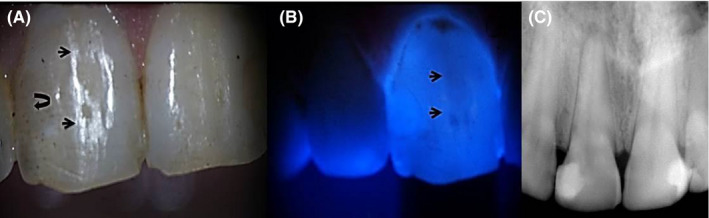
A, Clinical image; small arrows show crack line, curved arrow shows old resin filled restoration. B, Transillumination confirms crack line (arrow). C, Preoperative radiograph

A 23‐year‐old male dental student was referred to the Department of Endodontics, Faculty of Dentistry, Tanta University. The patient had no significant medical history. His chief complaint was pain associated with biting in the maxillary right central incisor. There was a Class III composite restoration in the distolabial aspect and superficial longitudinal crack in the labial wall of the crown. Transillumination showed a labial crack line (Figure 1A‐B) and cold test caused exacerbation of severe pain that remained after the removal of the stimulus. Gingival tissues were inflamed, and there was painful responsiveness to vertical percussion. However, radiographic examination showed radiolucency in the periapical region and the lamina dura was slightly widened (Figure 1C).

The case was diagnosed as irreversible pulp inflammation and the treatment plan involved RCT with management of the cracked crown using simvastatin. Details of the case and the treatment plan were discussed with the patient. He signed an informing consent for using simvastatin with the acknowledge of his understanding that this material will be used as intracanal medication and it was not previously used for this purpose. The patient agreed and permitted the publication of the case report.

**FIGURE 2:**
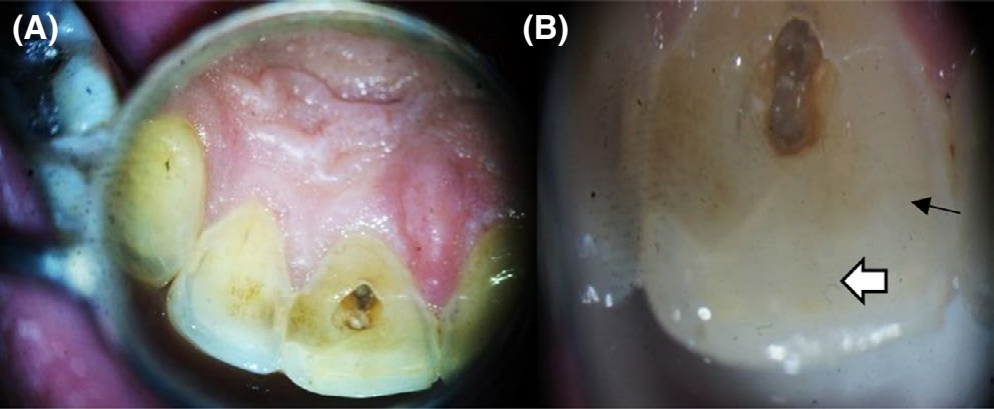
A, Access cavity with dark and contaminated labial wall. B, Old composite filling (white arrow) and the crack line appears form the preparation on the labial wall (black small arrow)

After local infiltration anesthesia of 2% lidocaine and 1:100 000 epinephrine (Lidocaine, Alex Pharma), the tooth was isolated with rubber dam and endodontic access was prepared following the conventional guidelines. A superficial crack was observed from the access preparation in labial wall that extended toward the incisal edge (Figure 2).

The working length was established with apex locator (Root ZX, J. Morita Corp.) at 1 mm from the radiographic apex. The canal was prepared with ProTaper Universal (Dentsply maillefer) files up to F4 using 2.5% sodium hypochlorite (NaOCl) as irrigating solution during instrumentation, and 17% ethylene diamine tetra acetic acid (EDTA) (META Biomed Co) as final irrigation to remove the smear layer. Apical patency was kept with a #10 file, and the canal was rinsed with normal saline and dried with paper points. Calcium hydroxide medicament (META Biomed Co.) was placed into the canal, and the coronal cavity was provisionally sealed with a temporary filling material (Cavit™ G, 3M ESPE).

**FIGURE 3:**
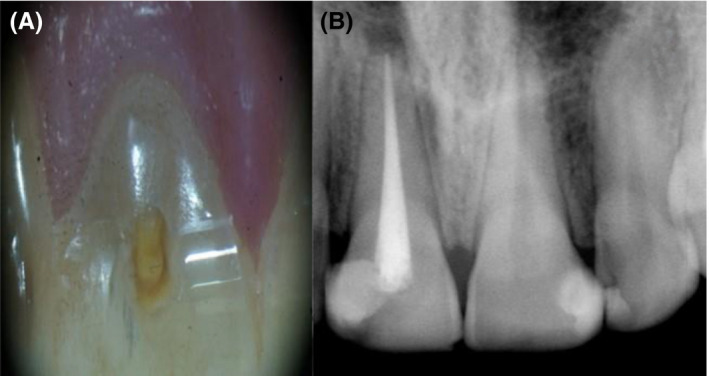
A, Access cavity after removal of simvastatin. B, postoperative radiograph

The patient was recalled after 1 week for removal of calcium hydroxide intracanal medication. He showed pain with filing at the coronal third of the labial wall. In spite of several trials of cleaning, the labial wall of canal preparation appeared dark, sensitive, and rough. After removal of calcium hydroxide, simvastatin was use as intracanal medicament (rest‐treatment). Three parts of simvastatin powder (Simvastatin_99%, HPLC, solid, Abcam) was mixed with one part of distilled water using a sterile metal spatula on a paper pad. When the mixture exhibited a thick creamy consistency, it was immediately carried on the master cone and inserted into the canal. The coronal cavity was then filled with Cavit™, and occlusal reduction was performed for elimination of occlusal contacts to avoid any overload or possibility of splitting the cracked tooth. The patient was kept at rest‐treatment for 3 months. Following that, simvastatin medication was completely removed, and the canal was freshly cleaned as previously mentioned. The canal was then filled with gutta‐percha and AH‐26 sealer (Dentsply, DeTrey, GmbH) using lateral condensation technique. A restorative glass ionomer filling (Prima Dental, GL2 2HA) was immediately used to seal the coronal preparation (Figure 3).

## 3 RESULTS

**FIGURE 4:**
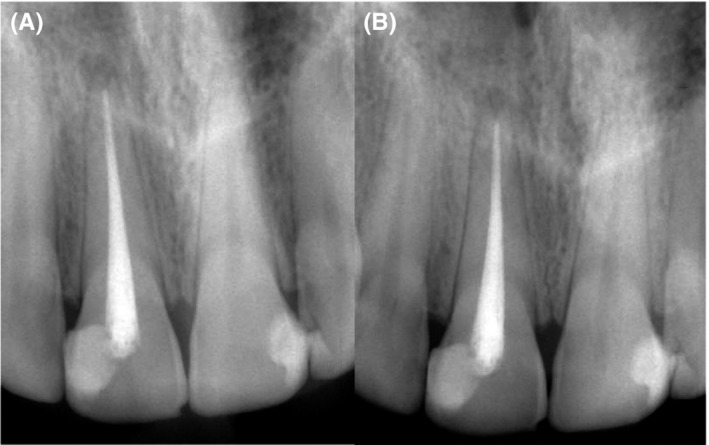
Follow‐up Radiographs (A) 6‐mo follow‐up (B) 12‐mo follow‐up shows complete healing of the apical lesion

The patient did not report any painful symptom during the rest‐treatment, and all signs and symptoms had completely disappeared on 1‐week follow‐up recall. On 6‐month follow‐up, the gingiva appeared normal and the radiograph showed normal periodontal ligaments with normal lamina dura (Figure 4A). On 12‐month follow‐up, the tooth was fully functioned without the need for full coverage with clinical and radiographic normal features (Figure 4B).

## 4 DISCUSSION

Tiny cracks are common and usually do not cause problems. In such cases, regular checkups are important to manage such problems in early stage. Various treatment modalities are available for advanced cracks (incomplete fracture). The choice depends on the location, direction, and extent of the crack. Cracks may be superficial, affecting the cusp of a tooth or deep to involve the root of the tooth. Some affect only the enamel; others may extend to the dentin or the pulp. Before treatment, reduction or elimination of occlusal contacts is essential to avoid an overload of a split tooth.^2^

Tenderness on biting and pain with cold, sweet, or hot or a combination of these are common complains of a cracked tooth. These symptoms can be explained by the hydrodynamic theory of pain first described by Feiglin^7^ and substantiated experimentally by Brӓnnstrӧm.^8^ This theory is based on the concept that rapid movement of dentinal fluid in the dentinal tubules causes pain. This movement stimulates mechanoreceptors in close proximity to the odontoblast cell body, which then activate delta nerve fibers (faster myelinated fibers), resulting in a short sharp pain.

Simvastatin is a new therapeutic material in the field of dentistry and still under dental research. Its structural analogs of HMG‐CoA (3‐hydroxy‐3‐methylglutaryl‐coenzyme A) that are considered to be the first line to control hyperlipidemia and it has been recognized as a safe and low‐priced drug with worldwide longtime usage.^9^, ^10^ Moreover, statin has multiple functions including anti‐inflammatory, antimicrobial, and induction of angiogenesis and improvement of the vascular endothelial cell function. In addition, several statins products have anabolic effect on bone metabolism ^11^ and promote bone formation through inhibition of both osteoclast activity and apoptosis of osteoblasts. And this effect appears to be related to increased expression of bone morphogenic protein‐2 in osteogenic cells.^12^

Crack sealed by dentin is considered the proper biological and mechanical management of tooth crack. Simvastatin was used in this clinical report as an intracanal medicament to seal the tooth crack because previous experimental studies proved that it can stimulate dentin formation.^13^, ^14^ Painful symptoms disappeared during the rest‐treatment which proved the therapeutic effects of simvastatin. The healing occurred in our study is in agreement with Madi et al,^15^ Rutledge et al,^16^ Jia et al,^17^ and Varalakshmi et al^18^; these studies used simvastatin for bone and dentin formation.

The exact mechanism or mode of action of simvastatin in the crack seal is not known. However, the anti‐inflammatory, antimicrobial, antioxidant, and wound/bone healing properties of simvastatin may explain healing and rapid relief of pain after application of the material inside the canal.

The use of simvastatin in the current case is considered as an empirical treatment that was based on experimental studies and further clinical studies should be conducted for investigation of its side effects and mode of action before its approval to be used in endodontics. Regardless the results of this case, the clinicians can apply this approach only when they have scientific evidence.

## 5 CONCLUSION

Fracture is one of the most common causes of tooth loss; therefore, it is important to avoid or eliminate risk factors which contribute to tooth fracture. The key factor is early diagnosis and treatment of the cracks so that they can be halted, or their progression slowed down. Simvastatin allowed a good management for a case suffered from cracked tooth.

## CONFLICT OF INTEREST

The authors deny any conflicts of interest related to this study.

## AUTHOR CONTRIBUTIONS

HA: involved in the study design, writing the manuscript, supervision, and critical review. MF: involved in conception, materials, and writing the manuscript. MS: involved in study design and writing the manuscript. SA: writing and critical review.

## ETHICAL APPROVAL

The patient signed an informing consent for using simvastatin with the acknowledge and understanding that the treatment material will be used as intracanal medication and it was not previously used for this purpose. The patient agreed and permitted the publication of the case report. The protocol was approved from the Department of Endodontics, Tanta University, Egypt.
